# Experimental research on surface acoustic wave microfluidic atomization for drug delivery

**DOI:** 10.1038/s41598-022-11132-9

**Published:** 2022-05-13

**Authors:** Qing-Yun Huang, Ying Le, Hong Hu, Zhi-jian Wan, Jia Ning, Jun-Long Han

**Affiliations:** 1 School of Mechanical Engineering and Automation, Harbin Institute of Technology Shenzhen, Guangdong, 518055 China; 2 Shenzhen Polytechnic, Shenzhen, 518055 China; 3 Department of Endocrinology and Metabolism, Shenzhen People’s Hospital (The Second Clinical Medical College, Jinan University; The First Affiliated Hospital, Southern University of Science and Technology), Shenzhen, 518020 Guangdong China

**Keywords:** Fluid dynamics, Asthma

## Abstract

This paper demonstrates that surface acoustic wave (SAW) atomization can produce suitable aerosol concentration and size distribution for efficient inhaled lung drug delivery and is a potential atomization device for asthma treatment. Using the SAW device, we present comprehensive experimental results exploring the complexity of the acoustic atomization process and the influence of input power, device frequency, and liquid flow rate on aerosol size distribution. It is hoped that these studies will explain the mechanism of SAW atomization aerosol generation and how they can be controlled. The insights from the high-speed flow visualization studies reveal that it is possible by setting the input power above 4.17 W, thus allowing atomization to occur from a relatively thin film, forming dense, monodisperse aerosols. Moreover, we found that the aerosol droplet size can be effectively changed by adjusting the input power and liquid flow rate to change the film conditions. In this work, we proposed a method to realize drug atomization by a microfluidic channel. A SU-8 flow channel was prepared on the surface of a piezoelectric substrate by photolithography technology. Combined with the silicon dioxide coating process and PDMS process closed microfluidic channel was prepared, and continuous drug atomization was provided to improve the deposition efficiency of drug atomization by microfluidic.

## Introduction

The earliest recorded pulmonary drug delivery process, also known as an approach in which drugs were delivered to the body through the respiratory tract, can be date back 4000 years. Drug delivery to the lungs mainly involves two different routes: one is the local action of the drug along the respiratory tract on the lung surface, the other is the route that the drug is transported throughout the body by the blood^1,2^. The former has increased significantly in the past 50 years as it has been recognized that even with relatively inefficient local treatments, the adequate dose level required is significantly reduced compared with oral/systemic administration, with relatively fewer side effects and toxicity problems. The large surface area, thin epithelial lining, and highly vascularized nature of the lung region itself make it an ideal choice for non-invasive drug administration. At present, inhalation therapy has been widely used to treat respiratory diseases, including asthma, chronic obstructive pulmonary disease (COPD), pulmonary and cystic fibrosis, and pulmonary hypertension^3–5^. Direct lung delivery via inhalation facilitates local targeting of the disease- or injury-specific region and allows for pain-free access where potential systemic side effects are minimized.

Asthma is a chronic inflammatory disease associated with excessive lymphocytes and eosinophils in the airway walls. This airway inflammation narrows the airways, causing a blockage or blockage of airflow during human breathing^6^. For inhalation therapy to be most effective, the drug-loaded aerosol must be deposited mainly in the inflammation part of the lung. Recent studies have shown that high eosinophil counts were found in the entire lung area in patients with certain forms of asthma, from the bronchi to the alveoli^7–9^. Therefore, direct deposition of drugs throughout the lower respiratory tract is considered the most effective treatment. One of the most critical factors affecting the lung deposition of particles or aerosols is the aerodynamic characteristics of particles, including the effect of particle or aerosol size, density, shape, hygroscopicity^10^. Many clinical studies have shown that aerosol particle size is critical to the efficacy of inhalation therapy. In general, the larger the aerosol size, the more likely it is to be deposited in the airway of the upper respiratory tract and even in the extrathoracic region. In addition, the aerosol with a small diameter is quickly exhaled during exhalation, and droplets smaller than 0.5 μm may be exhaled. At present, many studies have shown that 1–5 μm aerosol droplets are the best range for pulmonary drug delivery during normal tidal breathing^10–12^.

Compared with a traditional metered aerosol inhaler (MDI) and Dry Powder Inhalers (DPI), the atomizer can deliver more drugs due to a longer running time. In addition, unlike MDI, the atomizer does not require patient coordination skills, nor does it need to be driven by inhalation like DPI, which is urgently needed for those patients who have acute COPD or asthma attacks and can not self-medicate^13^. For the same reason, common inhalers have a poor agreement between children and elderly patients, but the dose in an atomizer can be adjusted with age, gender. Generally, clinical atomizers mainly include the following categories: pneumatic atomizers^6^, ultrasonic atomizers^14^, and vibrating mesh atomizers^15^. Nevertheless, these atomizers are limited by power requirements and sizes, hindering portable use outside hospital settings^16^. In addition, pneumatic atomizers have poor performance in producing aerosol monodispersity or narrowly dispersed aerosols, and ultrasonic atomizers are relatively large, also have limitations on size control, and molecules will be damaged during the ultrasonic atomization process due to the cavitation effect^17^. To avoid cavitation, it is necessary to reduce the power or to operate at higher frequencies where the power required to induce cavitation is lower than that required for the onset of atomization. More recent designs using meshes for atomization provide better portability, dose rate, and aerosol monodispersity, but these meshes are prone to clogging, which dramatically reduces throughput^18^.

**Figure 1:**
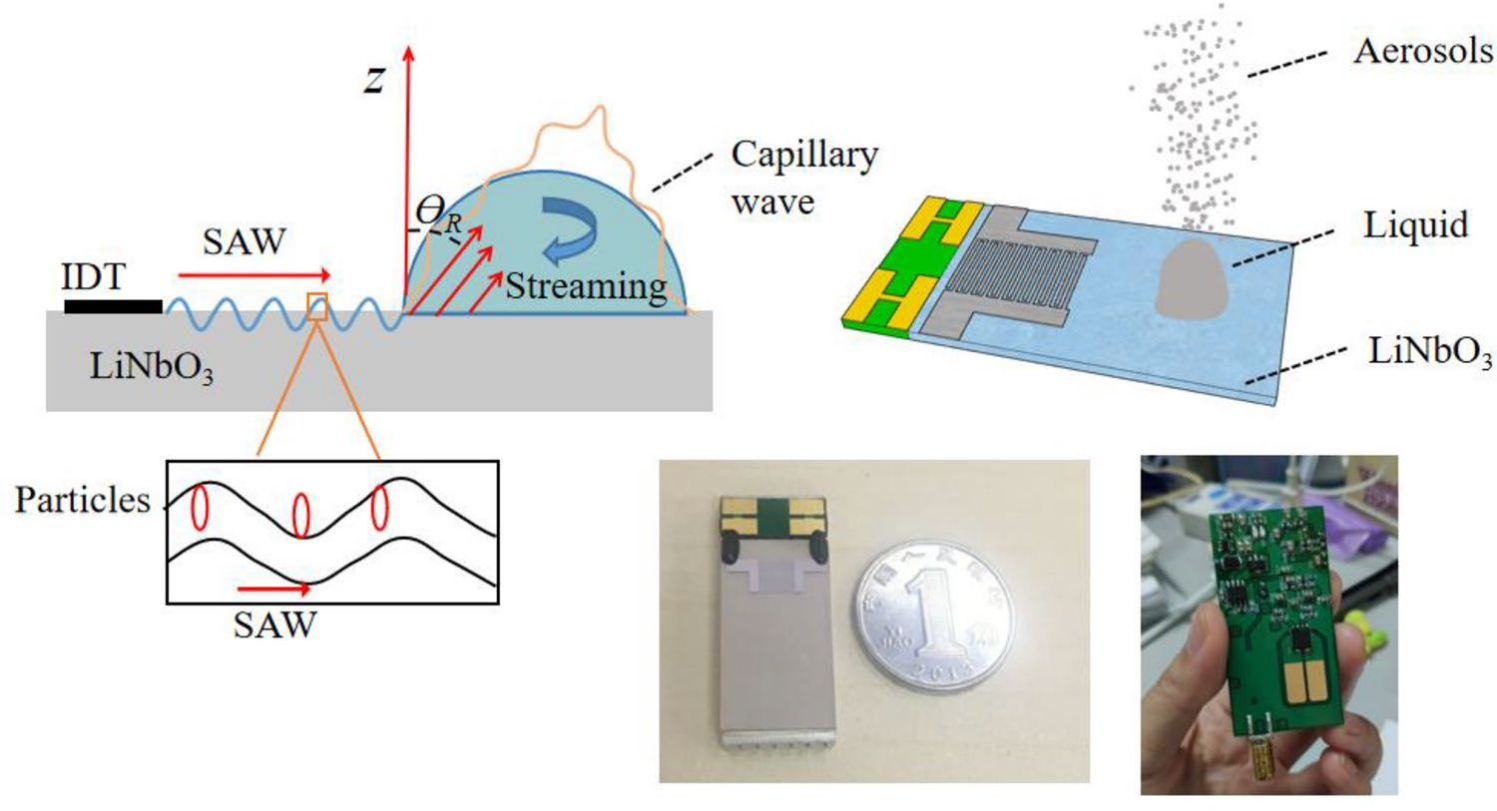
The top image shows a schematic diagram of atomization caused by SAW interacting with liquid. The bottom images show the piezoelectric substrate on which the SAW is generated (left) and portable battery-powered circuit used to generate the SAW (right). It uses three pieces of 3.7 V lithium batteries, and the maximum output of this driver circuit is about 110 Vp–p.

To solve all these problems related to the various delivery technologies listed previously. An alternative atomizers platform has been developed that employs the use of surface acoustic wave (SAW). SAW is a nanometer-order amplitude sound wave that propagates along the surface of a piezoelectric substrate^19,20^. Compared with conventional ultrasonic atomization (< 1 MHz), SAW atomization is driven at a higher frequency (> 10 MHz) and can generate a large amount of aerosol in a range of 1–10 μm. SAW is an efficient way to transfer mechanical energy into fluid^21,22^. Unlike ultrasonic waves in which energy is transmitted as a whole, the energy transmitted by SAW is localized on the substrate until it contacts with fluid. When a droplet is placed on the path where the SAW propagates, the SAW will attenuate into a leaky SAW (LSAW) mode upon its arrival at the boundary between the substrate and the liquid. Concomitant with wave attenuation is the leakage of acoustic energy into the droplet at an inclined angle called the Rayleigh angle. This process will cause an acoustic radiation pressure and circulating flow in the droplet, which is called SAW streaming, as shown in Fig. 1. According to the different conditions of the applied input power and SAW frequency, the following phenomena can be observed in the droplet: internal streaming, vibration, transport, and jetting, and atomization^23–25^. When the input power exceeds a certain threshold, enough energy is provided to the droplet's surface through fluid–structure coupling to overcome the capillary stabilization mechanism. Due to the perturbations caused by the massive acceleration of the substrate and the consequent streaming in the droplet, the capillary stress should no longer keep the gas–liquid interface in a stable form, and the interface ruptures to produce atomized aerosol droplets ^26–28^. Consequently, SAW atomizers can run at lower power of 2–3 W than conventional atomizers, which requires a power of above 10 W. At present, the corresponding driving circuit has been developed, and only three lithium batteries are needed for the power supply, as shown in Fig. 1, which shows the potential of this device in portable applications. For portable circuit-driven atomization details, please refer to the supplemental material video. Compared with the circuit drive in the previous literature^29^, the resulting atomization rate is greatly improved. It has also been shown that delicate proteins and even yeast cells could be atomized without damage by the acoustic irradiation arising from the SAW^22,30,31^.

An ideal atomizer can effectively deliver high doses of a drug and allow precise control of the droplet size distribution. In addition, SAW atomization applications have been proven to have a wide variety of applications, such as protein extraction^32^ and characterization for paper diagnosis^22^, drug delivery^12^, mass spectrometry^33^, spray cooling^34^. Anushi E Rajapaksa et al. reported that SAW hand-held atomizer produces droplets of pDNA with a size range suitable for delivery to the lower respiratory airways^31^. Layla Alhasan et al. demonstrated the SAW atomization platform as a promising method for efficient pulmonary stem cell delivery^4^. Thus, these studies established that SAW atomization could deliver drugs with droplet diameters 1–5 μm range to the deep lung region.

Nevertheless, current literature does not provide much information to quantitatively investigate the mechanisms underlying SAW aerosol generation, which was still unclear^35–37^. Collins et al. demonstrated that how the characteristics of the thin film, as a consequence, influence the size of the atomized droplets^35^. Winkler et al. studied the influence of the viscosity on the droplet size and the general atomization behavior^38^. Ju, Jungmyoung et al. investigated the deposited dry particle diameter distribution under high frequency. It was confirmed that as the frequency increases, the droplet size and the atomization speed are reduced^39^. The SAW frequency, in particular, has long been touted as a means to control the droplet size^35,37,39^. There was a consensus that increasing the device frequency can effectively reduce the size of aerosol droplets and satellite droplets, but the frequency value of capillary wave has always been controversial. Nevertheless, there appears to be some controversy surrounding the adoption of the subharmonic frequency proposed by Lang, i.e., the capillary wave frequency *f*_c_ is one-half of the excitation frequency *f*, many studies have also cited it to calculate the aerosol size^14,17^. This is due to the difficulty of accurately measuring the frequency at which capillary waves vibrate. Qi et al. has used advanced laser Doppler vibrometry to reveal the absence of any subharmonic excitation^21^. For destabilized capillary wave, the resonant frequency was either due to internal capillary-viscous resonance, *f*_c_ was given by ^21,40^

1 $$ f_{c} \sim \frac{\gamma }{\mu R} $$

Or internal capillary-inertia resonance, given by ^21^

2 $$ f_{c} \sim \left( {\frac{\gamma }{{\rho R^{3} }}} \right)^{1/2} $$

where *γ* is the surface tension, *μ* is the dynamic viscosity, *ρ* is the liquid density, *R* is the characteristic length scale. Since inertial forcing was usually confined below the viscous boundary layer *δ*, given by

3 $$ \delta \sim \left( {\frac{\mu }{\rho \omega }} \right)^{1/2} $$

where *ω* is the angular frequency, for water, substituting in Eq. (3), then the predicted value for the viscous boundary layer thickness was typically between 10^–6^ and 10^–7^ m. Since the characteristic thickness of the meniscus was more significant than the boundary layer thickness during continuous atomization, viscous effects dominate over inertial effects. Due to the viscosity, high-frequency excitation makes capillary waves possess oscillation frequencies of a relatively broad range around one order of magnitude but centered around the viscous-capillary resonant frequency. Sugiyama et al. studied the influence of the input power on the mist diameter were experimentally discussed. It was found that the average diameter of the mist does not depend on the input power, and the mist diameter was determined by the frequency^41^. Another study came to a different result: particle size and drop speed were increased by the input power rise^42^. Winkler et al., based on a fluid supply at the boundary of the acoustic beam via SU-8 microchannels produced by a novel one-layer/double-exposure photolithography method, investigated the aerosol distribution under different flow rates^43^. Therefore, these studies have shown that the parameters affect the aerosol particle size distribution. For an existing device and a specific fluid to be atomized, the input power and flow rates can directly regulate aerosol particle size distribution in real-time, which was essential for drug delivery efficiency. Additionally, as the frequency increased, the droplet size was reduced. However, the times required for atomization increased, which increased patient distress and inconvenience. Therefore, these two parts were contradictory, and a balance must be found. To date, Qi et al. use a supply needle to carry out the salbutamol drug delivery experiment, but while the drop was in contact with this supply structure, the SAW was transmitted into the structure, strongly affecting the atomization performance^12^. Additionally, no more experimental results were given for the deposition efficiency of drugs under different supply flow rates and frequencies. Moreover, it has not been possible to reach a clear conclusion about the drug atomization delivery efficiency in a microchannel, and the double exposure lithography method has strict requirements for lithography equipment. More specifically, the double photolithography process is relatively complex and expensive.

Based on these results, in this work, we demonstrate the use of a novel high-frequency acoustic atomization platform as an effective aerosolization technique for treating asthmatic patients. In particular, we reorganized the experimental atomization data to systematically analyze the effects of input power, device frequency, and flow rates on the aerosol particle size distribution. Droplet size measurements of atomized mist and visualization of the whole atomization process were made to understand the mechanism of SAW atomization aerosol generation. Based on the research results of these parameters and with the aid of a twin-stage impinger lung model, we then discuss the drug deposition efficiency under different fluid supply methods.

## Materials and methods

### Surface acoustic wave device and experimental setup

**Figure 2:**
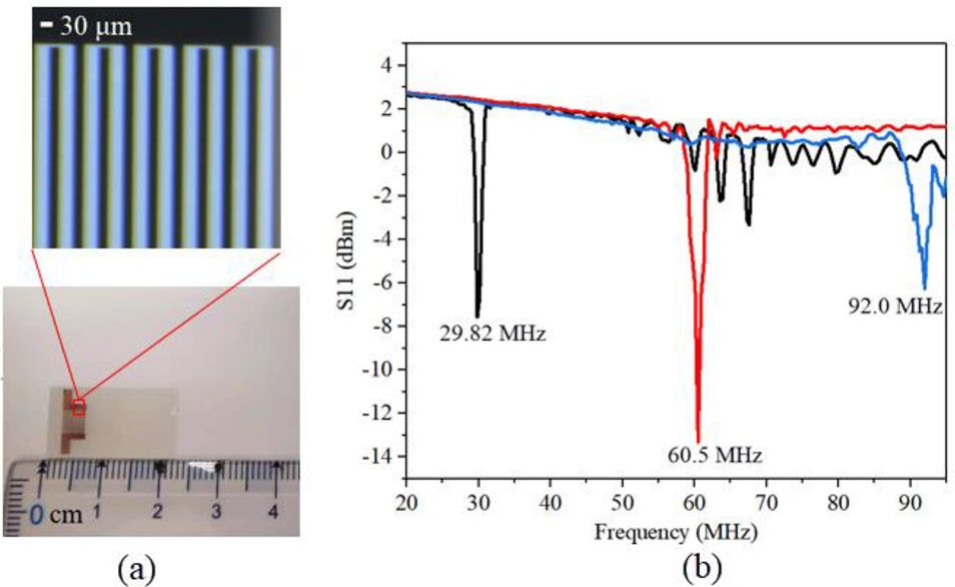
The device characterization (**a**) Image of the SAW substrate showing an enlargement of the IDTs for a 30 MHz SAW device. (**b**) Frequency response curves of SAW devices at different frequencies.

**Figure 3:**
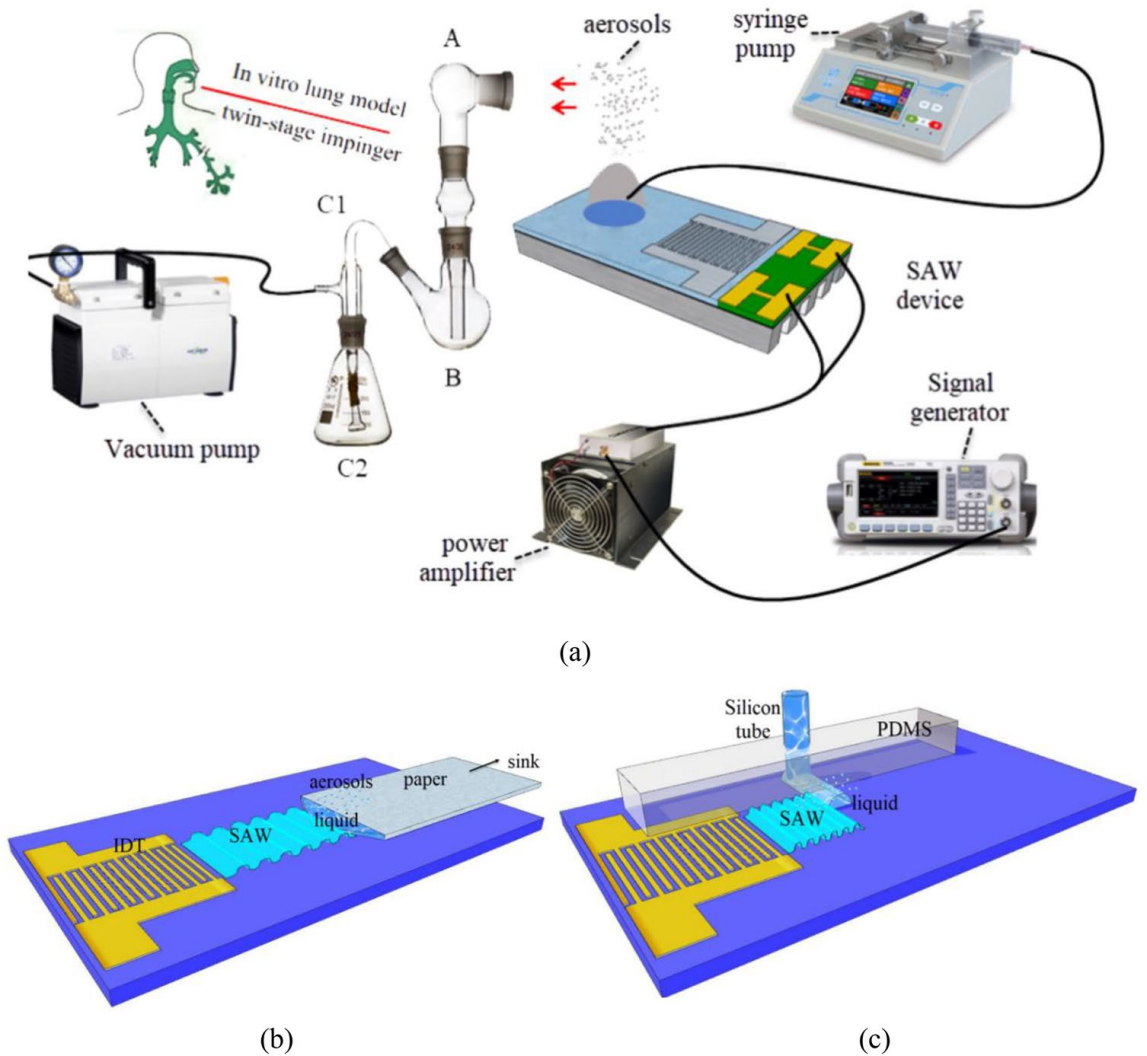
Schematic diagram of the glass twin-stage impinger lung model employed for the dose measurements. (**a**) schematic diagram of experimental device. (**b**) continuous atomization based on paper. (**c**) continuous atomization based on microchannel.

Briefly, the SAW device was fabricated on a single 128.68° Y-X LiNbO_3_ wafer with a thickness of 0.5 mm using the standard lithography and lift-off technology. Details of the fabrication procedures can be found elsewhere. The wafer containing the interdigital transducer (IDT) was subsequently diced into microchips, each with approximately 22 mm length and 12 mm width. The configuration of the IDTs comprised 30 pairs of straight fingers with a wavelength of 130 μm, 65 μm, and 43.3 μm. The gap and width of the IDT fingers are set to be a quarter of the SAW wavelength *λ*, and the apertures were all at 4 mm. The enlarged view of the 30 MHz device under the microscope was shown in Fig. 2a. The SAW device was placed on an aluminum metal heatsink to reduce any high power-induced temperature effects in all experiments, the heatsink measures 22 mm × 12 mm × 4 mm, using a thermal gel gap pad (RS component #7,074,764) assist in heat transfer between the two. The resonance frequency of the fabricated acoustic device were measured by a network analyzer (Agilent E5073C). Figure 2b shows the frequency response of the SAW devices at different frequencies. The SAW was generated by applying an RF signal to the IDT using a signal generator (RIGOL DSG3000) and amplified by a power amplifier (Mini-circuit LZY-22). The voltage across the SAW device, measured using an oscilloscope simultaneously (Wavejet 332/334, LeCroy, Chestnut Ridge, NY, USA). To ensure the relative stability of the free fluid surface during atomization, a polyester-cellulose cleanroom paper (C1; LymTech, Chicopee, MA) was used to siphon liquids from the sink to the substrate surface for continuous atomization, as shown in Fig. 3b. As such, we have chosen a Ventolin inhalation solution as the experimental material with a concentration of 2 mg/mL. The atomization phenomena induced by acoustic waves were recorded using a microscope (LW-FF 25 mmf/2.8 ULTRA MACRO 2.5–5.0X, LAOWA, China) with a CMOS camera (CP70-1HS-M-1900, Optronics, Germany) at a frame rate of up to 40,000 frames/s. A laser diffraction analyzer (OMCC, DP-02) was used to measure the atomized droplets' size distribution accurately. The droplet size distribution of the aerosol jet was measured for 90 s in a distance of 30 mm to the chip surface with a laser spot of about 20 mm in diameter. Droplet sizes were calculated using the Mie scattering theory. Room temperature and humidity were measured to be 23.5 °C and 72.5%, respectively. Droplet size measurements were made three times for the different experimental conditions to ensure consistency of results. Use a syringe pump (Refu, TYD01) to provide flow supply at different rates.

### Lung model and aerosol characterization

We aimed to demonstrate the feasibility and superiority of SAW atomization as a platform for treating asthma. A twin-stage glass impinger was used to conduct drug particle inhalation evaluation research by accepted China Pharmaceutical industry-standard in vitro model. Figure 3a shows the schematic diagram of the vitro model, and the glass impinger simulates an in vitro model of the human lung system. During the impact stage, it was filled with absolute ethanol to collect and dissolve salbutamol aerosol particles. It adds 30 ml of absolute ethanol to the second-stage conical flask C2 as the absorption solution and 7 ml of absolute ethanol to the first-stage distribution flask B as the absorption solution. Airflow of 60 L/min ± 5 L/min was drawn through the impinger during aerosol generation. At this flow rate, note that the aerodynamic diameter of the deposits in the A and B areas of the first stage was more significant than 6.4 μm. This part of the deposit was mainly due to the dominance of inertia in their motion, which represents the deposition of the upper respiratory tract of the human body. It was estimated that the aerodynamic diameter less than 6.4 μm was deposited in the second stage C1 and C2 regions, mainly due to gravity or diffusion motion, which represents the deposition of the lower respiratory tract. In this experiment, note that the dose deposited in region A represents the human oral cavity dose, region B represents the human throat dose, and C1 and C2 constitute the human lung dose. In addition, the theoretical dose represents the total volume of the drug solution delivered to the SAW device, while the volume atomized from the SAW device was defined as the emitted dose.

During the experiment, the volume fraction of each area was determined by comparing the dose, that is, the concentration of the salbutamol in each area with the total aerosol dose. The concentration and dosage of salbutamol were measured by UV spectrophotometer (UV-2450, Shimadzu, Japan). The determination of these dose fractions will be discussed in detail later on.

### Fabrication of SU-8 microchannel

**Figure 4:**
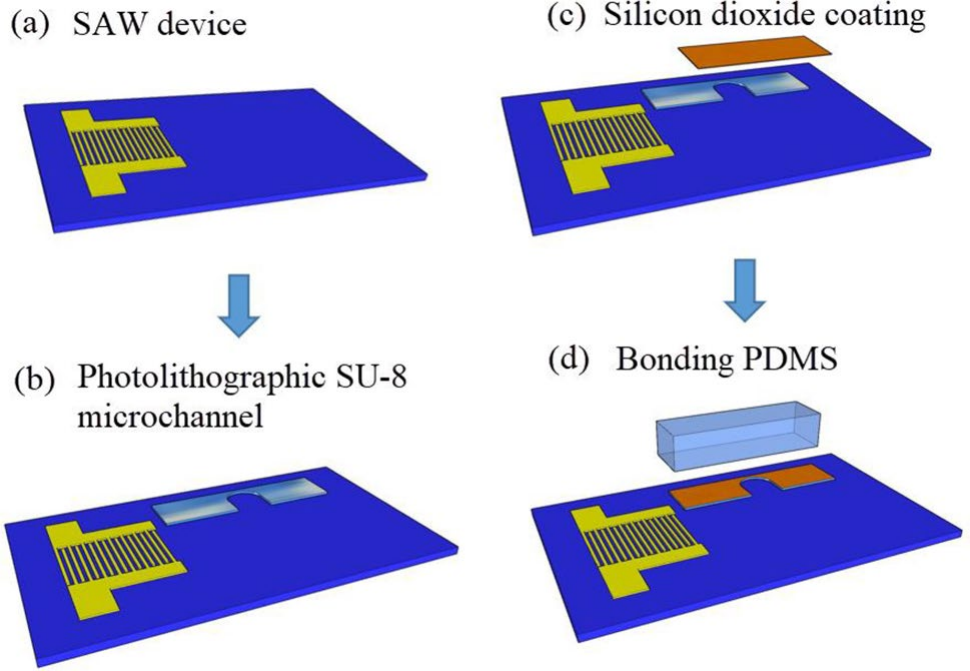
Schematic diagram of the fabrication scheme based on the SU-8 microchannel (**a**) SAW device. (**b**) Photolithography of SU-8 microchannels on the substrate surface. (**c**) Coating silicon dioxide film on the upper surface of the microchannel. (**d**) Bonding the PDMS layer with the microchannel to form a closed microchannel.

When the fluid volume on the SAW propagation path exceeds the film condition, that is, the characteristic fluid dimensions were more significant than the wavelength of the longitudinal wave, then the droplet ejection phenomenon will occur. Therefore, it was essential to reduce the characteristic fluid dimensions in the atomization zone when the input power and flow rate were determined. Note that the photostructurable epoxy SU-8 (Microchem Corp.) exhibits optical transparency, excellent chemical resistance, and high mechanical and thermal stability. It can be processed to have high microchannels with vertical sidewalls and aspect ratios, and the thickness that can be made was 10–500 μm. Figure 4 presents a schematic diagram of the fabrication scheme based on the SU-8 microchannel. This microchannel manufacturing process mainly includes three parts. The SU-8–2050 photoresist (Microchem Corp.) was spin-coated on the chip surface, forming a 50 μm thick layer. This thickness was consistent with the thin-film conditions at which atomization occurs at a frequency of 30 MHz, i.e. the acoustic wavelength in the liquid^44^, and subsequently prebaked at 95 °C on a hot plate for 5 min. The width of the microchannel was set to 500 μm. Using contact MDA-400 M UV exposure lithography machine for exposure, the exposure dose was 260 ml/cm^2^, and the exposure time was set to 10 s. After lithography, a soft bake at 95 °C was carried out for 7 min. The structure was finally developed in the developer solution for 7 min, and the chips were cleaned acetone and deionized water one by one, then dried in air. Followed by the coating process, a 500 nm thick SiO_2_ layer was sputter-deposited on the upper surface of the SU-8 microchannel. Then, a 2 mm thick polydimethylsiloxane (PDMS) thin layer and the substrate were put into the plasma together, the oxygen flow rate at 60 sccm was carried out, power was set to 50 W, and function time was set to 60 s. For a more detailed description of the PDMS production process, refer to the study of Wang et al.^45^. Finally, it was placed in an oven bake at 95 ℃ for 15 min to enhance the bonding further, the final structure was shown in Fig. 3 (c).

### Statistical analysis

The SPSS 19.0 statistical software was used to accommodate normally and non-normally distributed results. All results were presented as the mean ± standard deviation. A two independent samples t-test was used for comparisons between two independent groups. One-way ANOVA was used to compare multiple independent groups. The results were considered significant if *P* values were less than 0.05 (*P* < 0.05).

## Results and discussion

### Effect of input Power on aerosol size distribution

**Figure 5:**
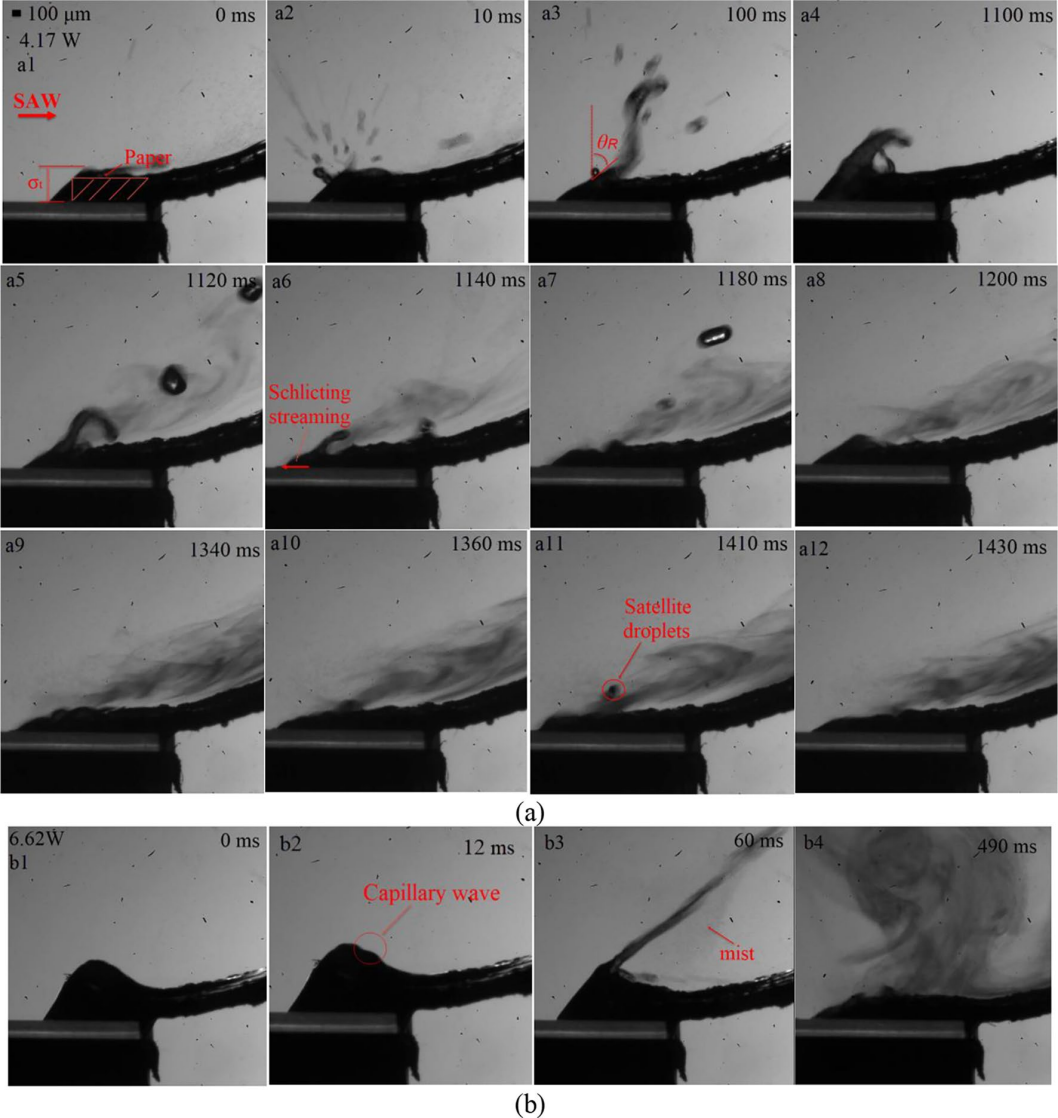
Continuous atomization process of liquid supplied by paper over time. The cross-hatched box indicates the position of the paper strip. (**a**) Atomization behavior with a SAW frequency of 30 MHz at a power of 4.17 W. (**b**) Atomization behavior with a SAW frequency of 30 MHz at a power of 6.62 W.

Figure 5 shows a high-speed sequence diagram of the continuous atomization behavior of salbutamol solution with a SAW device at a frequency of 30 MHz and different input powers. One end of the paper was connected with a sink, and the other was flat on the substrate surface. The entire atomization process mainly includes three stages: (a) the initial transient state at the beginning of excitation, (b) meniscus formation and deformation, (c) steady atomization process. In the initial stage, due to the siphoning effect at the front end of the paper, the liquid was absorbed from the sink to the substrate surface, and the liquid film began to accumulate. Simultaneously, the thickness of the liquid film (*σ*_*t*_) was similar to the thickness of paper, which was much larger than the acoustic wavelength in the liquid (*λ*_*f*_ ≈50 μm). Once the signal generator was started, acoustic energy coupling into the liquid in the direction of the Rayleigh angle drives the generation of Eckart streaming. In addition to the resulting acoustic streaming within the liquid, the acoustic radiation pressure also imparts a force at the interface, that together with the momentum transfer to the interface due to the Eckart streaming, exerts a body force on the liquid whose vertical component causes the interface into a sharp axisymmetric cone, forming multiple small crests on the free surface, and the threads were elongated and pinched off to produce droplets ejection, as shown in Fig. 5a1–a4. In particular, the jetting was formed in the inertial dominated regime when *σ*_*t*_ >  > *λ*_*f*_ . In addition, the duration of this stage has a great relationship with the siphon rate of paper and the input power, and it will be significantly shortened as the liquid film dimensions decreases or the input power increases^12^. As the jetting continues, the liquid film dimensions decrease. When *σ*_*t*_ was closed to or less than *λ*_*f*,_ capillary force dominated the regime compared to the inertial force. At sufficient input power, Schlichting streaming (boundary layer streaming) drives fluid motion within the boundary layer adjacent to the substrate through viscous shear, pulling a thin film out of paper towards the SAW irradiation, as shown in Fig. 5a6. The area pulled out by Schlichting streaming was called the meniscus area. During this process, the capillary wave destabilizes at the meniscus and subsequently breaks up into aerosols. As the atomization progressed, the liquid was continuously consumed, and negative pressure was generated at the meniscus so that the liquid was continuously siphoned from the sink through the paper strip to the surface of the substrate for the atomization process. When the flow rate in the paper matched with the atomization rate, the volume of the free liquid in the meniscus area remained constant, and a steady and continuous aerosol mist was generated on the surface of the meniscus, as shown in Fig. 5a9–a12. We also found that many satellite droplet jets were always mixed during stable atomization at this input power, as shown in the Fig. 5a11.

**Figure 6:**
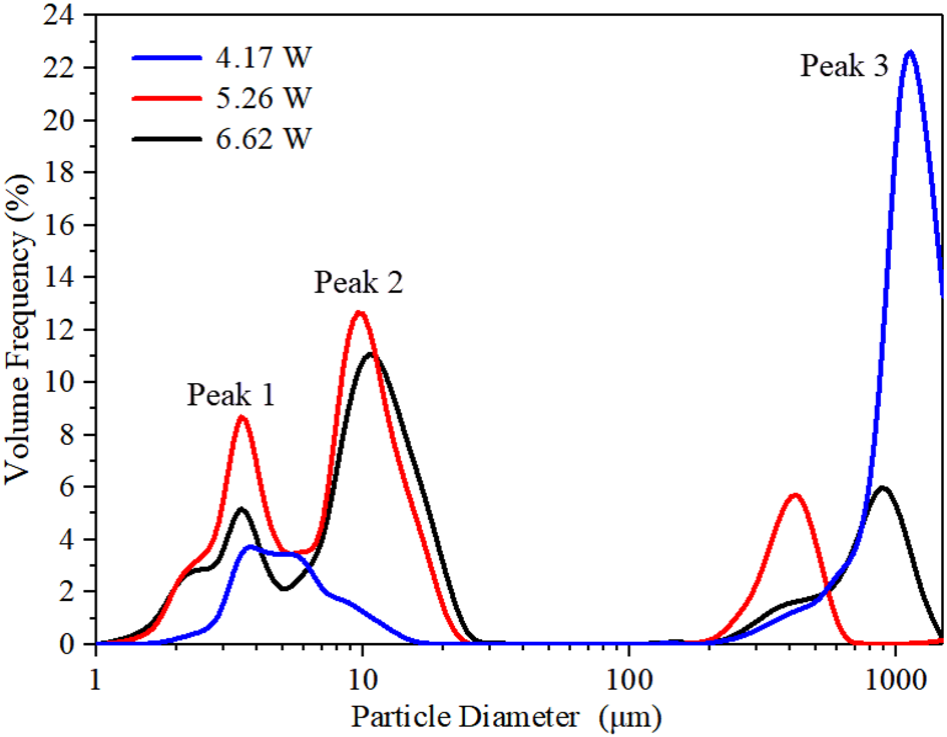
The aerosol size distribution of salbutamol solution atomized under different input powers.

Figure 6 shows the aerosol size distribution of salbutamol solution atomized under different input powers. It was observed that the total aerosol size measured results have a large distribution span, ranging from a few microns to one or two hundred microns, mainly including three peaks, which were concentrated around 3 μm, 10 μm, and 1000 μm. These peaks depend on the meniscus area formed at the tip of the paper strip. Large droplets of 1000 μm were produced from the satellite droplet ejection. It can be seen that under the action of 4.17 W input power, the most significant volume contribution was from the largest droplets. Interestingly, as the input power increased, the liquid was consumed rapidly due to the intense atomization, resulting in a thin front-running film head of the paper, as shown in Fig. 5b1–b4. This description was utterly consistent with the volume fraction changes of peak 3 in Fig. 6. Specifically, the volume fraction of particle size above 100 μm gradually decreased from 78.25% to 26.31% when the input power was increased from 4.17 W to 6.62 W. From the above results, it can be concluded that there is competition between jetting and atomization in the stage of the stable atomization process, which depends on the input power. Note that at low power, the required acoustic energy was dissipated by the accompanying jetting portion during atomization, resulting in a significantly weakened Eckart streaming and thus a significant drop in capillary wavenumber, which leads to the rise of both capillary wavelength and aerosol size. Therefore, it was essential to suppress these large droplets. The insights from the high-speed flow visualization studies revealed that this was possible by setting the input power above 4.17 W, thus allowing atomization to occur from a relatively thin film, forming a dense, monodisperse aerosol. Note that the first peak of 3 μm was produced from capillary waves due to Schlicting streaming, and the 10 μm order droplets were produced from capillary waves due to Eckart streaming effect, which is a steady vertical flow due to viscous attenuation. Both peaks originate from a thin front-running film of the meniscus, the ratio of these two peaks directly affects the volume fraction of droplets with diameter below 5 μm, which will determine whether a atomizer device is acceptable for asthma inhalation therapy (for this application, at least 30% is usually required). Table 1 shows the data of aerosol droplet size under different input power. From the distributions, three parameters were chosen to characterize the aerosol size distribution, *D*_v10,_ *D*_v50_ and *D*_32_. The former two parameters represent the 10%, 50% volume percentiles, The parameter *D*_32_ indicates that the Sauter mean diameter was the mean particle size related to the dose delivery efficiency when the particles were droplets of unit density^12,31^. In order to better understand the effect of input power on aerosol size, the total SAW power(*P*_SAW_)entering the liquid was calculated, and the related calculation process can refer to our previous study^27^.

**Table 1:** Data of aerosol droplets size under different input power.

Input power (W)	SAW power into water (W)	Aerosol droplets size		
		*D*_v10_/μm	*D*_32_/μm	*D*_v50_/μm
4.17	4.19	4.37	19.59	930.02
5.26	5.16	2.74	6.14	8.58
6.62	6.99	2.88	7.45	10.66
8.33	9.23	3.25	8.99	12.77

*D*_v10_—linear mean diameter.

*D*_32_—Sauter mean diameter.

*D*_v50_—Volume median diameter.

**Figure 7:**
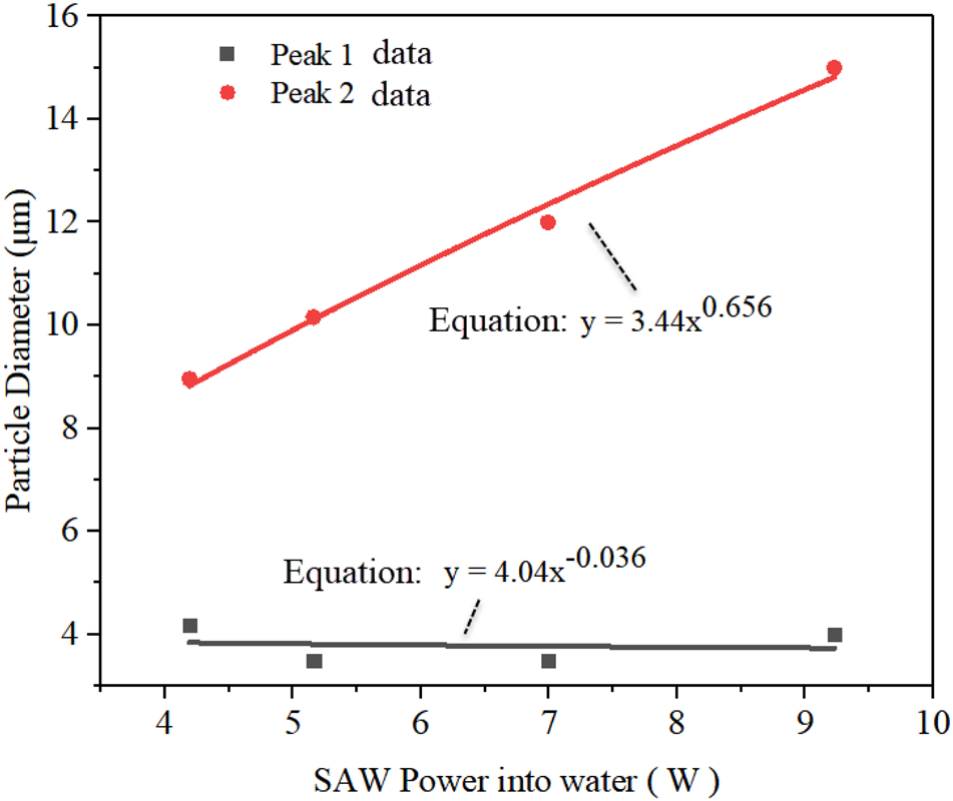
Experimentally measured aerosol size distribution under different SAW powers for each of the two peaks.

Note that the SAW power rise increased aerosol droplets size, this phenomenon can be explained by ultrasonic atomization theory, that is, increasing *P*_SAW_ directly caused an increase of energy absorbed by the liquid. This will increase the amplitude of the capillary wave generated on the surface of the liquid film, increasing aerosol droplet size and droplet velocity after atomization. Additionlly, increasing *P*_SAW_ drives stronger Eckart streaming effect in the film in the SAW propagation direction, leads to an increase in the acoustic body force, therefore producing shorter films, that, in turn, lead to larger droplets. Collins et al. suggested that thin film geometries determine the ejected droplet size, and given by ^35^:

4 $$ D\sim \frac{{\gamma H^{2} }}{{\mu L^{2} }}\frac{{We^{2/3} }}{f} $$

where *H* and *L* were characteristic height and length scales of film, *H/L* was close to the paper strip thickness, *We* = *ρL*(*u*_*0*_)^2^/*εγ* was an acoustic Weber number, where *u*_0_ was the vibration velocity of the SAW substrate. Figure 7 shows the experimentally measured average droplet size data under different SAW powers for each peak (i.e., peak 1 and peak 2).

It can be seen that under the SAW power of 5.16 W, the linear average diameter *D*_v10_ was 2.74 μm, and the mean droplet size *D*_v50_ was 8.58 μm, respectively. Similarly, the volume of the liquid film in the meniscus area remained constant. Note that as the *P*_SAW_ increases, the data points of peak 1 basically maintain a constant trend, which was directly reflected in the diameter changes of *D*_v10_. Therefore, Schlicting streaming has not been enhanced during this change. A simple power function can be obtained to describe the relationship between Peak 2 data points and *P*_SAW_, i.e., *y* = 3.44*P*_SAW_^0.656^_._ It was indicated that the peak 2 data increased linearly according to the *P*_SAW_^2/3^ trend. Since *P*_SAW_ ~ *ρu*_*0*_^2^, implying that *We* ~ *P*_SAW_. Therefore, the mean droplet size produced by SAW atomization *D*_v50_ ~ *P*_SAW_^2/3^, consistent with the results in Table 1. Particularly, at higher power, the film length was approaching the limit due to the increase of atomization intensity, and the influence of further increased power on droplet size gradually decreased.

### Effect of device frequency on aerosol size distribution

**Figure 8:**
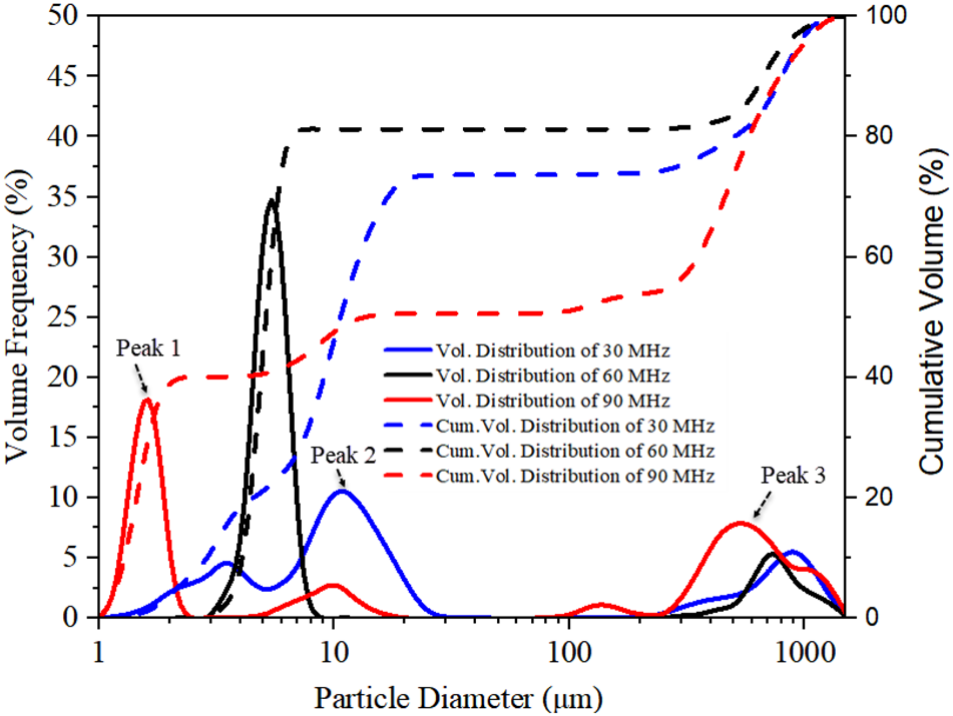
Aerosol droplet size distribution of salbutamol solution atomized at 6.62 W and different frequencies, measured by laser diffractometry. Each frequency value was tested three times and then the results were averaged. Note that the mean diameters *D*_v50_ for the three analysed frequencies were 10.66 μm, 5.13 μm, and 3.02 μm, respectively, and the the standard deviation were 1.298, 1.584, and 0.889, respectively.

**Figure 9:**
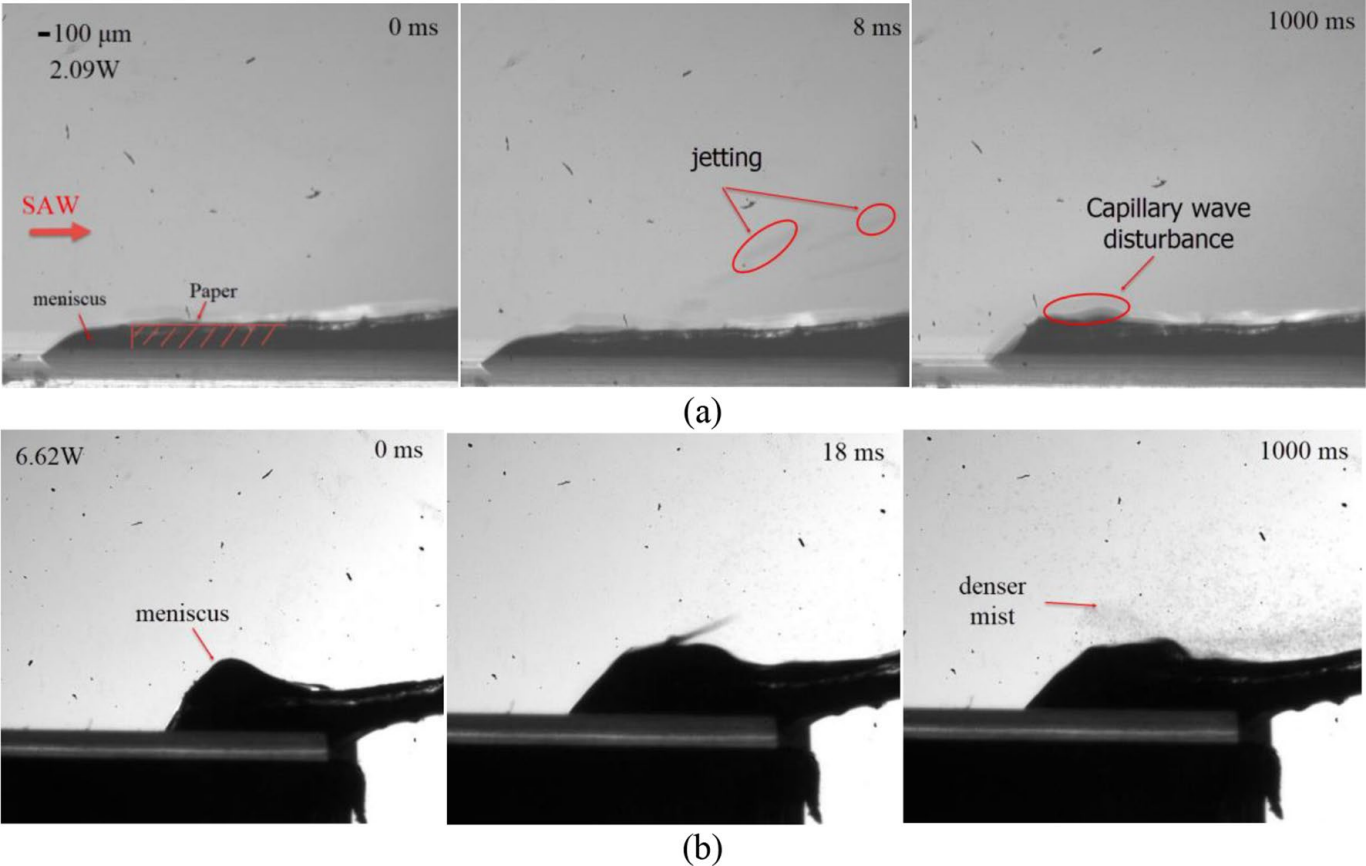
Atomization process at different input power with working frequency of 60 MHz. (**a**) 2.09 W; (**b**) 6.62 W.

Figure 8 shows the aerosol size distribution results of salbutamol solution atomized under different device frequencies at 6.62 W input power. To obtain accurate results, each frequency value was tested three times, and the results were averaged. The mean diameters *D*_v50_ for the three analyzed frequencies were 10.66 μm, 5.13 μm, and 3.02 μm, respectively. With the increase of device frequency, the mean diameter of aerosol droplets produced by atomization decreases gradually, consistent with previous studies ^39^. For a frequency of 30 MHz, the prominent peak was around 10.16 μm, and the secondary peak was observed in measurements of about 2.88 μm. As the input frequency increases to 60 MHz, the initial double peaks merge into a single one located at 4.8 μm, at the same time, a third peak located above 100 μm increases. Interestingly, this change can be explained that by the streaming Reynolds number. The streaming Reynolds number is expressed as follows^46^:

5 $$ {\text{Re}}_{s} = \frac{\rho \nu R}{\mu } $$

where *v *is the streaming velocity inside the liquid (ca. 10^–1^–10^–2^ m/s, measured by using particle image velocimetry (PIV) method, can refer to our previous study ^19^), *R* is the characteristic height of liquid film (ca. 10^–3^ m), *μ* is the liquid viscosity (salbutamol solution, ca. 10^–3^ Pa.s), *ρ *is the density of the liquid. According to the previous literature^46^, the mechanism that allows the cascade of subharmonics from high excitation frequencies to low capillary wave frequencies was the formation of turbulence in the acoustic streaming induced induced by surface acoustic waves within the fluid bulk. When the value of Res is above the critical value of ~ 10^2^, acoustic streaming is turbulent, which predicts the transition of turbulent flow to subharmonic and period-doubling cascades, leading to result in chaotic flow behavior. For salbutamol solution, *ρ* = 1.03 × 10^3^ kg/m^3^, *μ* = 1 × 10^–3^ Pa.s, In the case of frequency of 30 MHz and input power of 6.62 W, the streaming velocity *v* was 180 mm/s; Substituting these parameter values into Eq. (5), then the predicted value for the streaming Reynolds number was 185. This also shows that the turbulent acoustic streaming induces bulk flow within the liquid and drives the generation of capillary waves through shear before atomization occurs. At this time, the Eckart streaming effect becomes dominant, resulting the most significant volume contribution of the middle peak. Note that for a 90 MHz device, the streaming velocity was reduced to 50 mm/s, since the higher the frequency, the less SAW energy entering the liquid. In this case, the predicted value for the Reynolds number can be obtained to be 50, the acoustic streaming failed to reach the given threshold of turbulence. Also, as the SAW amplitude decreases, the capillary wave response transforms from a low-frequency broadband cascade to a single peak at *f*_SAW_, which directly leads to a gradual increase in the proportion of peak 1. This is consistent with previous studies (Qi et al., 2008, Blamey.J et al., 2013). From the above results, the Schlicting streaming gradually dominates in the high-frequency atomization process. However, the proportion of peak 3 increases slightly due to the ejection of much finer droplets from the film. For a frequency of 60 MHz, with an input power of 2.09 W, atomization was often challenging to initiate even though significant capillary waves and surface vibrations can be observed on the liquid film surface, as shown in Fig. 9a. As the input power increases to 6.62 W, at 1000 ms, the generated mists were more intensive than at low power, as shown in Fig. 9b. The generated mist appeared much finer, compared to those at 30 MHz, when comparing Fig. 9b with those in Fig. 5b. Specifically, for a frequency of 60 MHz, under a power of 6.62 W, the liquid temperature at the paper edge was 85 ℃, while the temperature of the aerosol produced was only 35 ℃. This is essential for the protection of drug properties. For a more detailed experimental description of the thermal effect during liquid atomization process, please refer to our previous study^27^.

The insights from the high-speed flow visualization studies revealed that the minimum threshold power values required for continuous paper atomization at each frequency were 2.09 W, 2.63 W, and 3.31 W, respectively. For a 6.62 W input power, the atomization rates of water at 30 MHz, 60 MHz, and 90 MHz were 6.35 μl/s, 2.31 μl/s, and 1.33 μl/s respectively. For detailed experimental data of atomization rate, refer to our previous study^27^. It has to be remarked that, although increasing the device's frequency can effectively reduce the size of aerosol droplets, the rate at which liquid is atomized is also decreased. Thus, there was an operation trade-off. In order to obtain optimal 1–5 μm aerosol droplets size for deep lung penetration and maximum drug delivery, a higher device frequency was favored. On the other hand, it was ideal that SAW devices administer a given volume of drug in the shortest possible time. These two parts were contradictory, and a balance must be found, which will be confirmed by measurements with the lung model later on.

### Effect of the liquid flow rate on aerosol size distribution

For an existing device and a specific liquid to be atomized, none of these parameters can be changed in situ, that is, during device operation. Therefore, for a given setup, liquid flow rate and input power were two parameters that can control the aerosol size in real-time. To analyze the aerosol droplet size distribution variation with the liquid flow rates, measurements were obtained atomizing with constant input power to eliminate the input power dependence. During the atomization process, there is a critical flow rate. When the liquid flow rate marginally above the critical flow rate, the substrate surface can be completely wetted and atomized to avoid parasitic heating of the substrate. Refer to the ultrasonic atomization theory, the critical liquid flow rate Q_crit_ was calculated as follows^40^

6 $$ We = \frac{{fQ_{crit} \rho }}{\gamma } $$

where *We* is the acoustic Weber numbers, *f* is SAW frequency, *γ* is the surface tension, *ρ* is the liquid density. Note that the critical flow Q_crit_ is independent of the SAW power and is related to the threshold at which atomization begins to occur. The atomization process will discontinue when the liquid flow rate is less than the critical flow rate Q_crit_. The increase in the temperature of the substrate causes the liquid to boil, which changes the local refractivity of the particle size analyzer, so that accurate detection results cannot be obtained. This part will be explained in detail later. According to previous studies ^35^, atomization begins when the Weber number approaches a critical value of 1. That is, there is sufficient inertial stress to overcome the surface tension of the liquid. It was proposed that by equating the inertial forcing to stabilizing capillary stresses i.e. by taking *We* equal to unit, that is

7 $$ Q_{crit} = \frac{\gamma }{f\rho } $$

**Figure 10:**
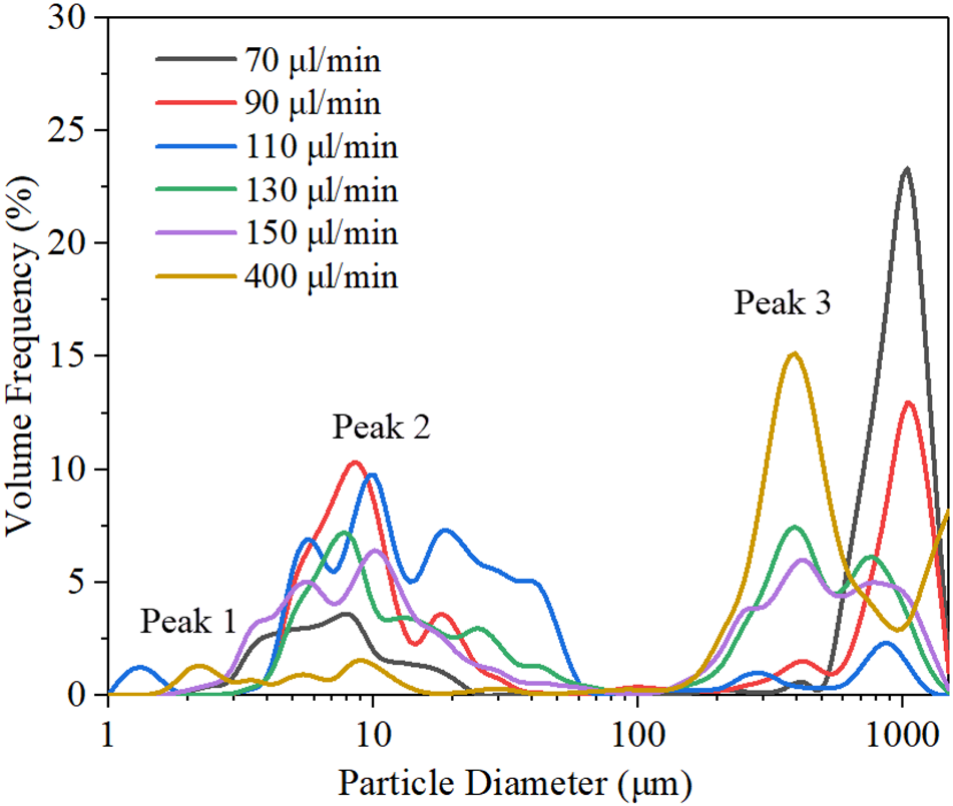
Aerosol droplet size distributions measured by laser diffractometry for different liquid flow rates with an SAW frequency of 30 MHz and input power of 6.62 W. Note that the Sauter mean diameter for the six analysed flow rates were 21.17 μm, 13.51 μm, 9.54 μm, 10.58 μm, 12.79 μm, and 32.51 μm, respectively.

This is the minimum critical flow rate above which the film's sufficient thickness is available to cover the working areas all time. Thus, the energy was utilized to the maximum. For a frequency of 30 MHz, substituting the parameter values of salbutamol solution into Eq. (7), the predicted value for the critical liquid flow rate Q_crit_ was 43 μl/min. The upper bound of the maximum flow rate was closely related to the atomization rate. When the maximum flow rate was more significant than the atomization rate, the liquid film began to accumulate and no longer meet the film condition, i.e., the conditions for atomization. For a 6.62 W input power in a 30 MHz device, the atomization rate was about 381 μl/min. Figure 10 shows the aerosol size distribution for different liquid flow rates. Note that, when the flow rate was increased from 70 to 400 μl/min, the Sauter mean diameters D_32_ appear to decrease first and then increase. It can be seen that at the flow rate of 70 μl/min and 90 μl/min, the volume contribution of peak 3 was 74.59% and 43.37%, respectively. Associated with an increase in the flow rate, the volume contribution of this part gradually decreased. It has to be remarked that if the flow rate was too low for sufficient thickness of a film to be maintained on the substrate surface, intermittently, the working surface gets exposed to air. Thus, a low flow rate will render the insufficient supply of fluid and the discontinuous atomization state. This was also concluded in earlier studies of Wink et al.^43,44^. Under the action of 6.62 W input power, at this point, it must be noted that the temperature of the substrate exceeds 150 °C. At this point, the liquid produced large droplets in the form of rapid boiling. The significant volume contribution of peak 3 origins came from this, which causes a change in the local refractivity in the aerosol.

**Figure 11:**
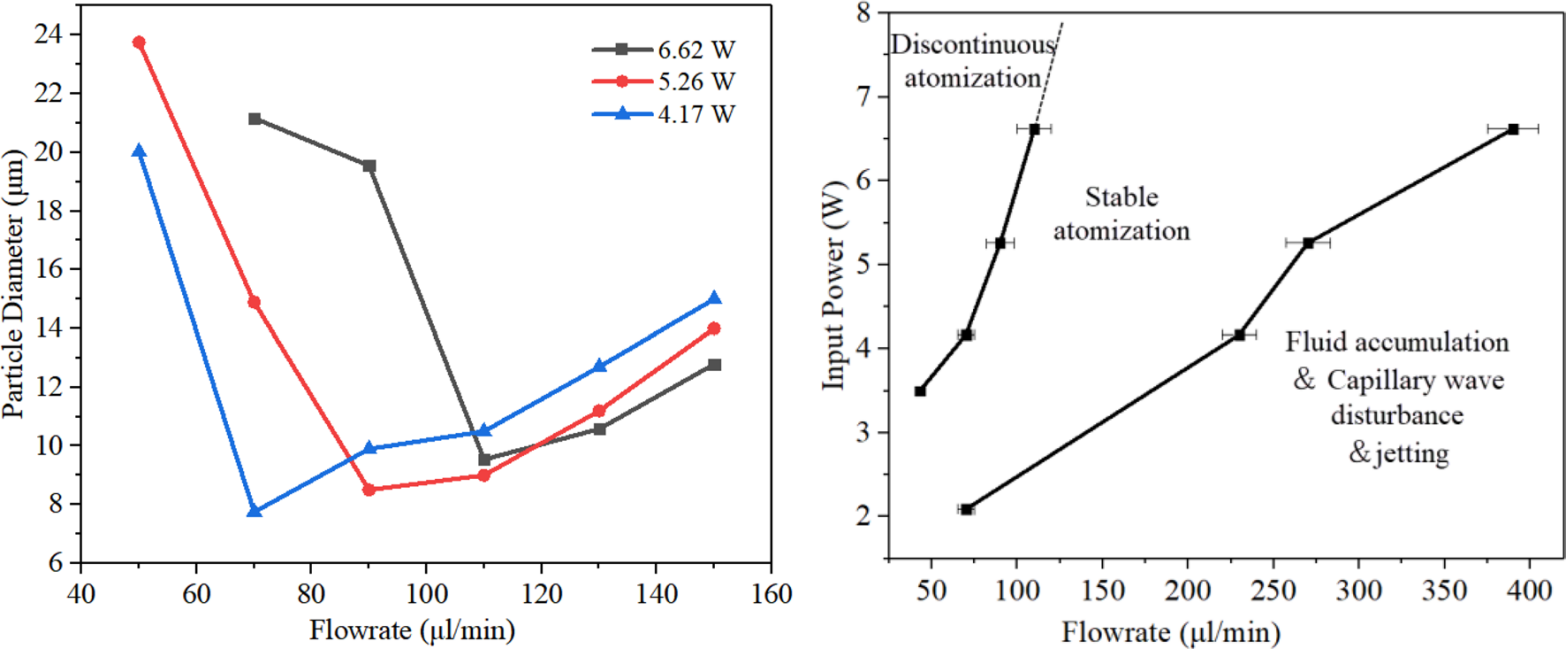
Effect of flow rate and input power on aerosol droplet size: (**a**) aerosol droplet size changes versus input power and liquid flow rates. (**b**) three different atomization regimes were determined by the combination of input power and flow rates.

When the flow rate reached 110 μl/min, the atomization was stable, and the aerosol size distribution presented a multi-peak mode. At this time, the volume contribution with aerosol droplet size greater than 100 μm accounts for 9.94%. When the flow rate reached 400 μl/min, the liquid accumulated on the SAW propagation path, and then the atomization stopped. Similarly, note that the dominant inertia regime and the ejection occurred. Figure 11a shows the effect of flow rates and input power on aerosol droplet size. According to experimental measurements, we found that the Sauter mean diameters can be effectively changed by adjusting the input power and liquid flow rate to change the film conditions. For a 5.26 W input power with a 90 μl/min flow rate, the Sauter mean diameter D32 was 8.5 μm. Interestingly, note that the combination of input power and flow rates can define three different atomization regimes, i.e., discontinuous atomization zone, stable atomization zone, and liquid film accumulation area, as shown in Fig. 11b. When the liquid flow rate exceed the atomization rate, results in fluid accumulation, accompanied by capillary wave disturbance and and no atomization occurs. Low flow rates will make the atomization process discontinuous as the atomization rate far exceeds the rate of fluid supplied to the substrate. Significant heating was observed in the case of a discontinuous atomization regime, especially using high SAW power. Since heat generation was representative of underutilized mechanical energy, it will cause local temperature rise, device damage and affect the SAW device's overall efficiency^47,48^. Therefore, the best aerosol droplet generation can be obtained when atomization behavior is located in the stable continuous atomization zone.

### Dose measurements and comparison

**Figure 12:**
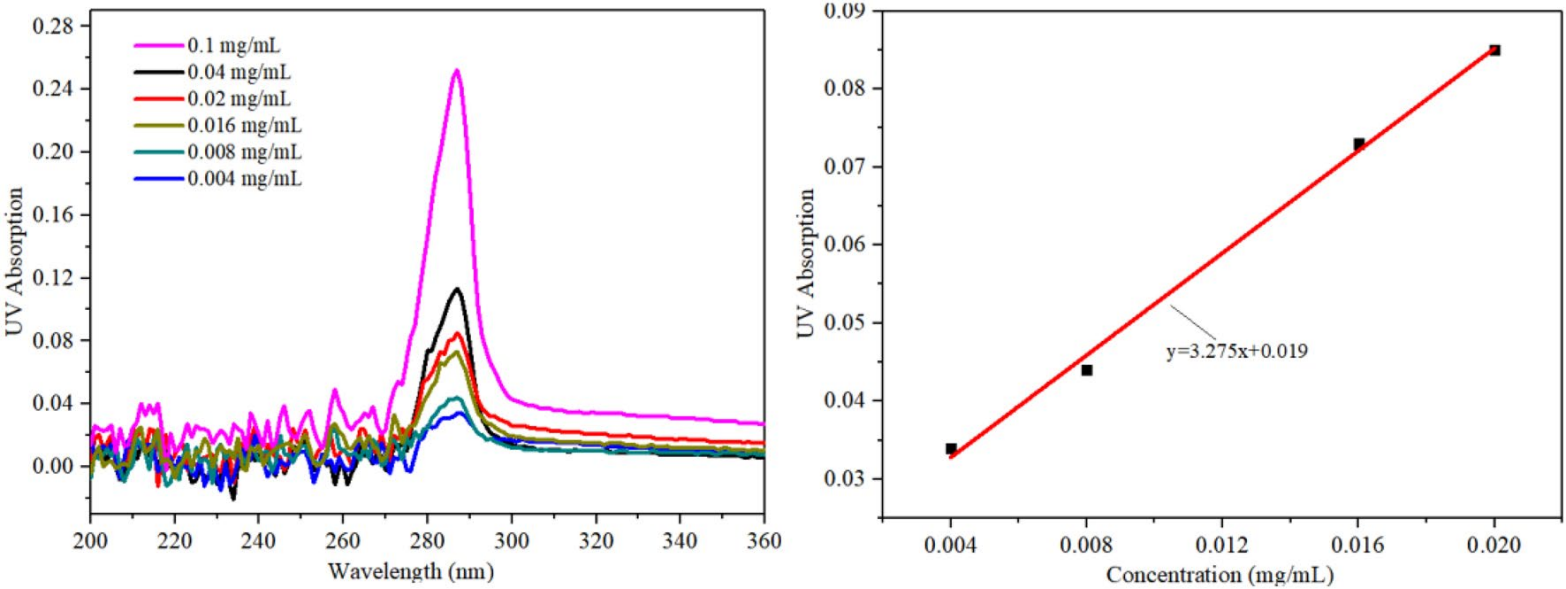
(**a**) UV absorption of salbutamol with different concentrations. (**b**) calibration curves based on the absorption peaks obtained in (a) used for the dose measurements from the different areas in the glass impinger.

Figure 12a shows the UV absorption curve of the standard salbutamol solution. Note that the salbutamol solution mainly absorbs UV light 287 nm. As the concentration increases, the absorption peak gradually increases, and the UV absorption of salbutamol has a linear fitting relationship with the concentration, as shown in Fig. 12b. We atomized the salbutamol solution and collected aerosols in each stage of the glass impinger. By comparing the measured UV absorption against the calibration curve, we can get the salbutamol concentration in each area in the glass impinger.

In inhalation therapy, the aerodynamic behavior of the droplets (controlled by Stokes' law) was crucial as an important indicator to measure the quality of inhaled drugs. The data recorded represent the physical diameters of the droplets can be converted into equivalent aerodynamic diameters using the following relationship^12^

7 $$ D_{a} = \left( {\frac{\rho }{{\rho_{0} }}} \right)^{1/2} D $$

where *D*_a_ and *D* are the aerodynamic and the physically measured diameters, *ρ* is the density of the solution, and *ρ*_*0*_ ≡1 g/cm^3^. In order to evaluate the effect of SAW atomization technology on the deposition of salbutamol in each area of the glass impinger, an experimental study was carried out based on the current physical aerosol particle size research results. It was worth noting that the proportion of drug particles entering the lung area during the experiment was called the acceptable particle dose (FPD). This parameter will determine whether an atomizer is suitable for asthma inhalation therapy and delivery efficiency. Table 2 shows the deposition of salbutamol atomized inhalation in each area with a frequency of 30 MHz, an input power of 5.26 W, and a flow rate of 90 μl/min. Each set of experiments was repeated three times.

**Table 2:** The deposition of salbutamol in each area measured by the two-stage impinger model.

Area	Aerodynamic diameter (μm)	Salbutamol particle collected (mg)
Oral area ( A)	> 6.4	0.75 ± 0.1
Throat area (B)	> 6.4	0.12 ± 0.1
Lung area (C1 + C2)	< 6.4	0.53 ± 0.08
Emitted salbutamol dose		1.4 mg ± 0.1
FPD < 6. 4 μm		0.53 mg ± 0.08

**Figure 13:**
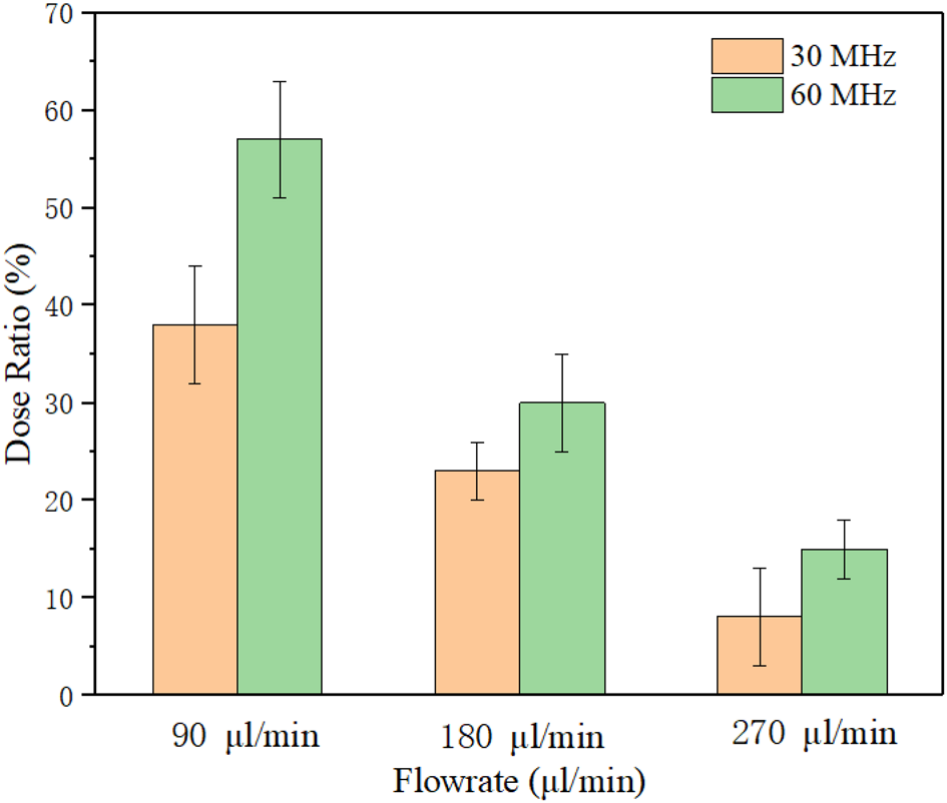
Dose rate of salbutamol particles deposited in lung area at different flow rates.

It can be seen from Table 2 that the deposition rates of oral cavity A, throat B, and lung C were 53%, 9%, and 38%, respectively. Experimental results also revealed that the emitted dose generated by SAW atomization accounts for 70% of the theoretical dose. The remaining (< 30%) drug loss was mainly due to thermal effect precipitation on the device substrate, which is in complete agreement with the literature results^12^. Increasing the input power can increase the atomization rate, but it will raise the aerodynamic size of the drug, enhance the thermal effect, and then reduce the efficiency of the SAW drug delivery device. Figure 13 shows the deposition rate of the lung area at 5.26 W input power with different device frequency versus different flow rates. Note that under the action of a frequency of 30 MHz, the deposition rate of the lung area gradually decreases with the increase of flow rate. When the flow rate reaches 270 μl/min, it exceeds the atomization rate of 266 μl/min under 5.26 W input power^27^. At this time, the deposition rate of salbutamol particles in the lung area was only 8.1%. This was consistent with the accumulated volume fraction of the aerosol size less than 6.4 μm measured by the laser diffractometer at the same input power, as shown in Fig. 8, which verifies the accuracy of the experimental scheme. It should be noted that the flow rate of 90 μl/min was already the minimum flow rate under the action of this power. If the supplied rate continues to decrease, it will lead to the discontinuous state of atomization. Simultaneously, reducing the flow rate will increase the atomization time, which will cause various discomfort for patients, so it was challenging to meet the requirements of drug atomization inhalation. At a frequency of 60 MHz and flow rate of 90 μl/min, the deposition rate reaches 57%. In this case, the thermal effect of the device was reduced, the corresponding SAW administration efficiency was improved, and more than 80% of the drug could be delivered into the respiratory system. If the device frequency was increased to 90 MHz, the atomization rate was only about 60 μl/min at the same power, which was challenging to meet the requirements of atomization inhalation. However, increasing the atomization rate requires increasing the input power, which was accompanied by a series of problems such as further heating the device and the increase of aerosol drug particle size, so this part of the experiment did not continue. Therefore, we can conclude that the device should be operated at a frequency of 60 MHz, an input power of 5.26 W, and a flow rate of 90 μl/min, such that the dose ratios across the various criteria were maximized.

**Figure 14:**
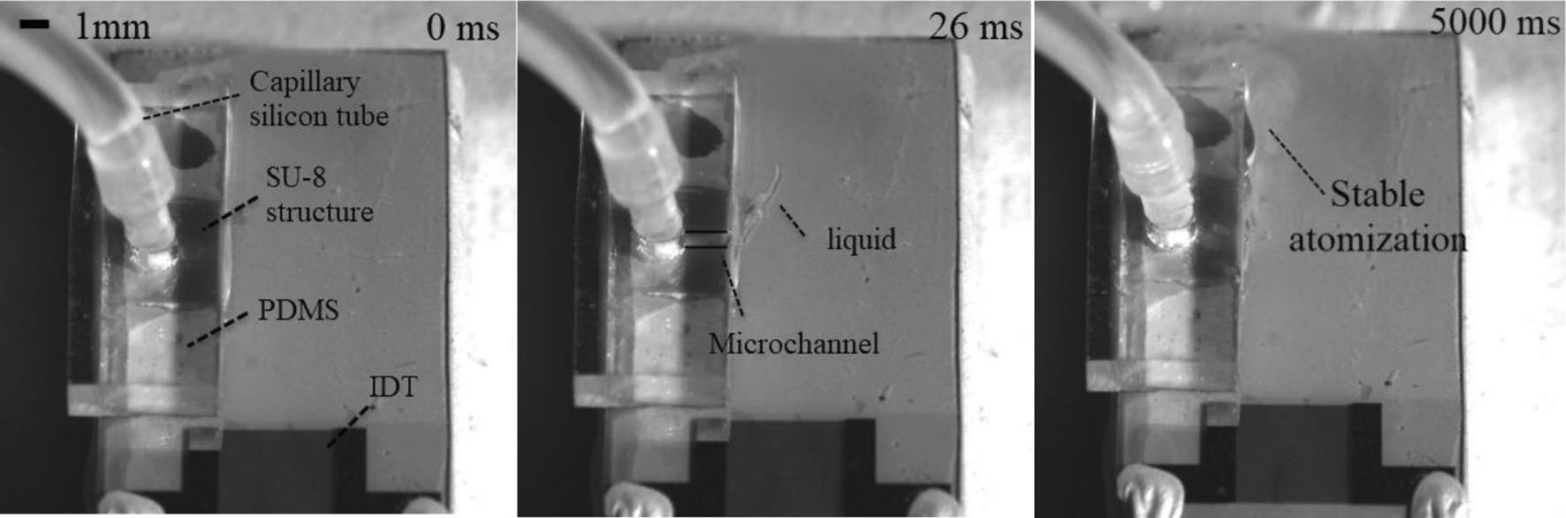
High-speed camera captures images of SAW atomized salbutamol solution based on SU-8 microchannel fluid supply with an SAW frequency of 60 MHz and input power of 5.26 W.

**Figure 15:**
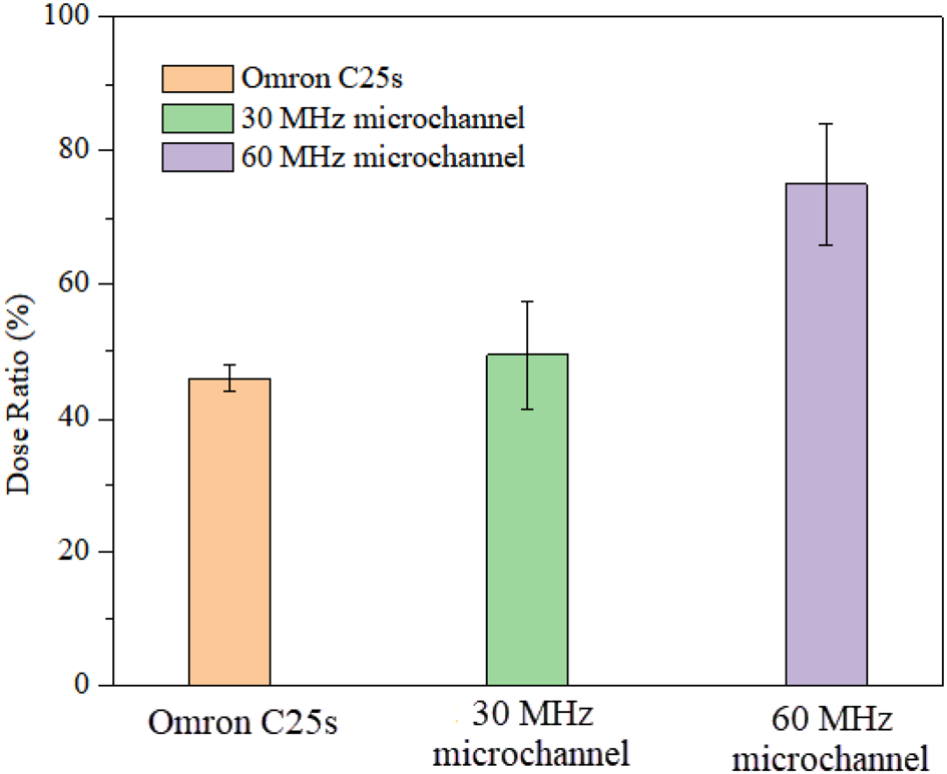
The lung deposition rates obtained by atomizing salbutamol solution using a medical atomizer and a SAW atomizer. Note that the SAW atomized salbutamol solution based on SU-8 microchannel fluid supply with a flow rate of 90 μl/min and input power of 5.26 W.

As SAW excites the liquid and destabilizes the liquid–air interfacial boundary equilibrium, the liquid spreads into a thin film as a precursor to the start of atomization. A thin film liquid is more accessible to atomize than a drop-shaped liquid because less viscous energy will be lost, and more energy can reach the surface of the liquid to produce capillary waves. In previous research^26,35^, the mist size strongly depends on the thickness and volume of the liquid on the SAW device. However, due to the limitations of the paper itself, it was unrealistic to use a smaller thickness of paper for the flow. Therefore, the fluid supply via on-chip polymeric microchannels prepared by photolithography directly on the chip surface was a good choice. These related processes have been introduced in Sec.IIC above. Figure 14 shows the high-speed camera captures images of SAW atomized salbutamol solution based on SU-8 microchannel fluid supply at supply at a frequency of 60 MHz, an input power of 5.26 W and flow rate of 90 μl/min. We can identify the dynamically stable fluid film at the exit of the microchannel at the boundary of the acoustic beam. Note that the salbutamol solution was rapidly actuated and atomized under the excitation of SAW, and we found that the ejection of large satellite droplets was not observed within 10 s. Figure 15 shows the lung deposition rates obtained by atomizing salbutamol solution using a Omron C25s medical atomizer and a SAW atomizer. Note that the SAW atomized salbutamol solution based on SU-8 microchannel fluid supply with a flow rate of 90 μl/min and input power of 5.26 W. It can be seen that in the inhalation experiment of the Omron C25s medical atomizer, the deposition rate in the lung area was 46%, which was much higher than the deposition efficiency of traditional DPI (about 19%) and MDI (about 29%)^49^. Note that compared with Omron medical atomizer, the deposition rate of using a SAW atomizer with a frequency of 60 MHz was significantly improved (*P* < 0.01), the deposition rate can reach 75%. Simultaneously, there was no statistically significant difference in lung deposition rate between Omron medical atomizer and SAW atomizer with a frequency of 30 MHz (*P* > 0.05). From the standpoint of dosage alone, the results show that SAW atomization technology based on microchannel fluid supply was a strong competitor of drug delivery technology on the market. Additionally, the unique advantages of SAW devices, such as small size, low cost, low power consumption, and no noise, coupled with accurately designed and dedicated electronics as well as signal solutions, solve the miniaturization issue of radiofrequency signal sources, which makes SAW atomization technology can become an alternative to these conventional atomizers. Moreover, the drive circuit for SAW devices has been developed, and only three pieces of 3.7 V lithium batteries are needed to supply power, which shows the potential of this device for portable applications. For portable circuit-driven atomization details, please refer to the supplemental material video.

## Conclusions

This study demonstrated that SAW atomization can produce suitable aerosol concentration and size distribution for efficient inhaled lung drug delivery and is a potential atomization device for asthma treatment. We used a SAW device to conduct an experimental study on aerosol size distribution. The effect of input power, device frequency, and liquid flow rate on aerosol size distribution during atomization was investigated by multiple experimental tests. The results revealed that operation power was also an important parameter, and the input power may change atomization characteristics. As the power was increased, the liquid was consumed very rapidly due to the intense atomization. The liquid dimensions at the meniscus were therefore tiny, resulting in fewer droplets ejected. The insights from the high-speed flow visualization studies revealed that this is possible by setting the power above 4.17 W, thus allowing atomization to occur from a relatively thin film, forming dense, monodisperse aerosols. Furthermore, in the case of steady and continuous atomization, increasing the input power will enhance Eckart streaming in the liquid film and then cause the aerosol droplet size to increase, suggesting that the aerosol droplet sizes increased linearly according to the *P*_SAW_ ^2/3^ trend. With the increase of device frequency, the aerosol droplet size produced by atomization decreases gradually. We also demonstrated that the Schlicting streaming gradually dominates in the high-frequency atomization process. According to experimental measurements, we found that the aerosol droplet size can be effectively changed by adjusting the input power and liquid flow rate to change the film conditions. Significantly, due to the precise characteristics of the fluid supply system, it can effectively reduce the jetting phenomenon during the atomization process. Our studies also show that the device should be operated at a frequency of 60 MHz, an input power of 5.26 W, and a flow rate of 90 μl/min, such that the dose ratios across the various criteria were maximized.

Using our new fluid supply approach, we atomized salbutamol solution to fine droplets in an almost ideally shaped aerosol plume, and there was no jetting phenomenon during this process. Using SAW device atomized salbutamol solution based on SU-8 microchannel fluid supply, the deposition rate in the lung area can reach 75%, significantly more significant than the 46% typically achieved with a medical atomizer. Notably, there was a significant statistical difference between each other (*P* < 0.01). From the standpoint of dosage alone, SAW atomization technology based on microchannel fluid supply was a strong competitor of drug delivery technology on the market. Therefore, these results lend confidence to the attractiveness and feasibility of the SAW atomization platform as a true miniaturized and integrated handheld platform for portable inhalation therapy. It can be widely used in the treatment of asthma and COPD.

## Supplementary Information

Supplementary Video 1.

Supplementary Video 2.

Supplementary Video 3.

## Data Availability

All data and materials are described in the manuscript.
